# Environmental stability of a uranium-plutonium-carbide phase

**DOI:** 10.1038/s41598-024-56885-7

**Published:** 2024-03-17

**Authors:** Barbara Etschmann, Owen P. Missen, Steven D. Conradson, Stuart Mills, Yang Liu, Joël Brugger

**Affiliations:** 1 School of Earth, Atmosphere & Environment, Monash University, Melbourne, Australia; 2 Geosciences, Museums Victoria, Melbourne, VIC Australia; 3 Department of Chemistry, Washington State University, Pullman, WA USA; 4 Department of Complex Matter, Josef Stefan Institute, Ljubljana, Slovenia; 5 Monash Centre for Electron Microscopy, Monash University, Melbourne, Australia; 6 Present Address: Centre for Ore Deposit and Earth Sciences (CODES), University of Tasmania, Hobart, Australia

**Keywords:** Environmental sciences, Materials science

## Abstract

A plutonium-rich carbide, (U,Pu)(Al,Fe)_3_C_3_, was discovered in a hot particle from the Maralinga nuclear testing site in South Australia. The particle was produced between 1960 and 1963 and has been exposed to ambient conditions since then. The new phase belongs to a group of ternary carbides known as 'derivative-MAX phases'. It formed at high temperature within an explosion cloud via rapid eutectic crystallisation from a complex Al–Fe–U–Pu–C–O melt, and is the major Pu host in this particle. Despite signs of volume expansion due to radiation damage, (U,Pu)(Al,Fe)_3_C_3_ remains highly X-ray crystalline 60 years after its formation, with no evidence of Pu leaching from the crystals. Our results highlight that the high-energy conditions of (sub-)critical explosions can create unexpected species. Even micro-particles of a derivative-MAX phase can effectively retain low-valence (metallic-like character) Pu under environmental conditions; the slow physical and chemical weathering of these particles may contribute to the slow release of radionuclides over decades, explaining constant low-levels of radionuclides observed in fauna. This study further suggests that rapidly quenched eutectic melts may be engineered to stabilise actinides in nuclear waste products, removing the need for hydrometallurgical processing.

## Introduction

Understanding the deportment of plutonium (Pu) and uranium (U) in the environment is necessary in order to determine how these radionuclides may be mobilised and affect living organisms, and to design effective mitigation and/or remediation strategies^1^. This information also helps assessing the long-term stability of radioactive waste disposal facilities. The long-term fate of Pu and/or U-particles depends on the nature of the source material and is dictated by their formation mechanism, the release conditions, the nature of the phase hosting the actinides, and the environment in which they were deposited^2–5^.

A putative U–Pu-carbide phase was recently identified in a hot particle from the North-east plume associated with the Taranaki Test Site at Maralinga, South Australia. This particle, referred to as ‘Bruce’, was most likely from the Vixen-B sub-critical nuclear tests^6,7^, a part of the British nuclear weapon testing program conducted in Australia in 1952–1963. The Vixen-B trials (1960–1963) were ‘safety’ tests designed to investigate the performance of nuclear components subjected to a crash or a fire. This involved the use of conventional explosives (TNT) to detonate Pu-containing nuclear warheads, which resulted in over 22 kg of ^239^Pu being scattered over the area^8^. The U–Pu-carbide phase was identified on the basis of semi-quantitative Energy Dispersive Spectrometry (EDS) data showing U, Pu, Al, Fe, and C as major components, and X-ray Absorption Near-Edge Structure spectroscopy (XANES) results showing the presence of low valence (metallic-like) U and Pu^6^. Uranium, and by inference Pu, in carbide phases have metallic-like electronic structures as demonstrated by Butorine et al.^9^ using a combination of high-energy-resolution-XAS and the Anderson inclusion model.

**Figure 1:**
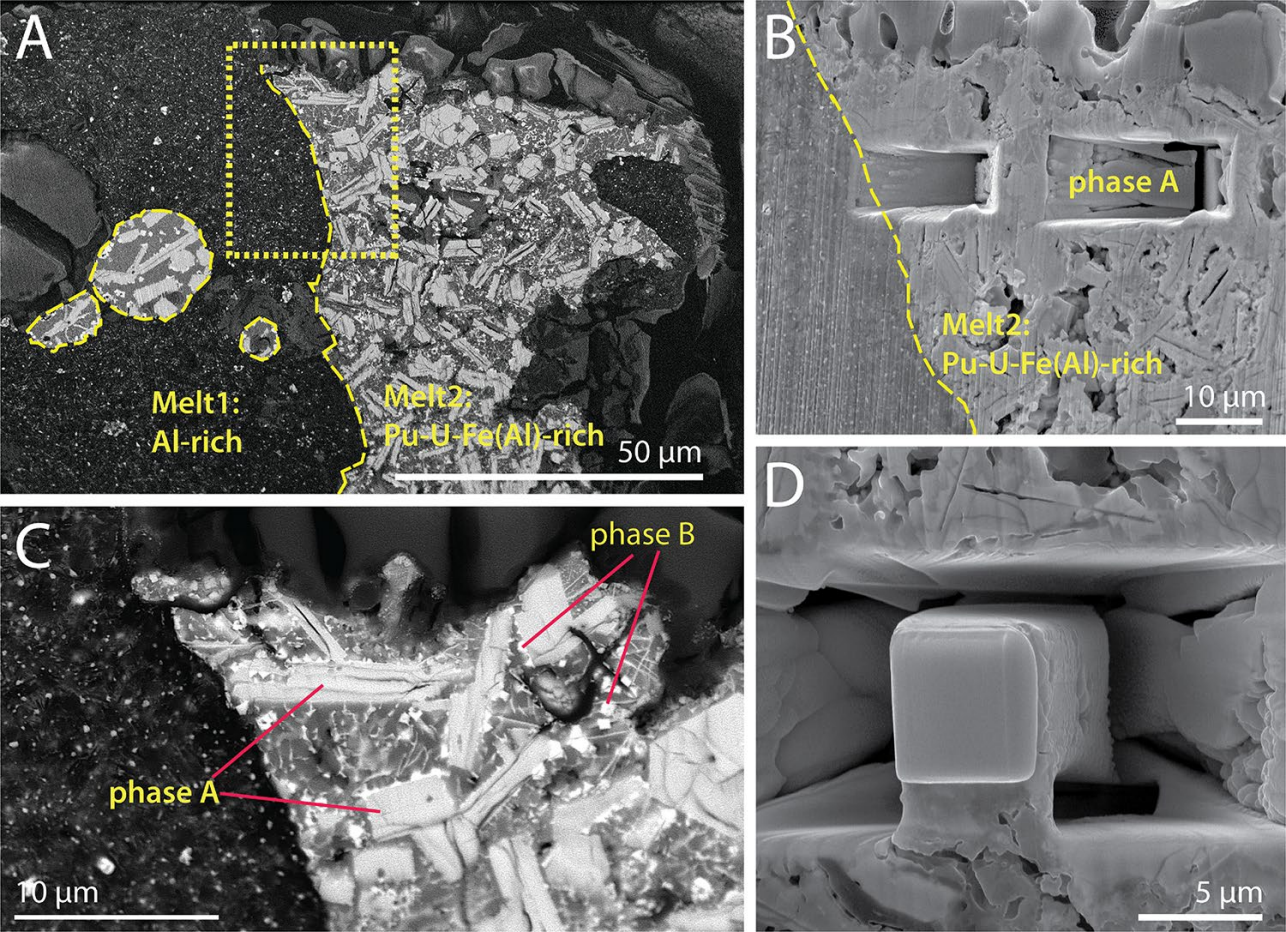
Microtextures within Bruce (**A**, **C**) and FIB-SEM cut (**B**, **C**). The particle consists of two immiscible melts, Melt 1 is Al-rich; Melt 2 is Pu–U-Fe ± Al-rich (**A**). Phase A (**C**) is the main Pu-carrier in the particle. The crystal targeted for extraction for the single crystal analysis is in the area shown in the box in (**A**); the trenching is illustrated in (**B**), and the crystal before lifting in (**D**).

Uranium- and Pu-carbides are usually pyrophoric at µm-grain size, i.e. they oxidise rapidly in contact with oxygen or water^10,11^; yet this particle survived in the regolith for ~ 30 years under near-surface semi-arid conditions before being collected by remediation crews in the 1980s^7,8^. Thereafter, the particle was stored under ambient conditions until it was examined with synchrotron radiation in October 2018, and sliced open with a focused ion beam (FIB) in May 2019, exposing the U–Pu-carbide phase (Fig. 1A). Further FIB-scanning electron microscope (FIB-SEM) investigations in March 2020 highlighted that this Pu–U-carbide phase showed no signs of oxidation upon direct exposure to atmosphere for ~ 10 months.

As described by Cook et al.^6^, Bruce is a complex particle, with textures indicative of formation via cooling of at least two immiscible polymetallic melts that formed in the explosion environment: Melt1 is Al-rich, and Melt2 is Pu–U–Fe(Al)-rich; Melt2 often forms globules within Melt1 (Fig. 1A). During eutectic cooling (e.g. Fig. 1 in Derimow and Abbaschian^12^), Melt2 first crystallized a U–Pu–(Al)–C phase (Fig. 1C), labelled Phase A by Cook et al.^6^. These acicular crystals account for ~ 50 vol% of bulk Melt 2, and carry most of the Pu in the Bruce particle. They are decorated by small (≤ 1 µm) cubic-shape grains of another putative Pu–U-carbide; finally, the matrix consists of an Fe–Al-rich alloy.

To understand the nature of the main Pu carrier phase in the Bruce hot particle, and the reason for preservation of the low-valence state of U and Pu in this particle, we used FIB-based techniques to extract a single crystal, and synchrotron single-crystal X-ray diffraction to determine its crystal structure.

## Crystal structure of Phase A: ternary (U,Pu) carbide

In June 2022, a FEI Quanta 3D FIB-SEM was used to extract a ~ 32 µm^3^ ‘chunk’ of phase-A from Bruce (Fig. 1B,D). This sample was stored under ambient conditions for 3 months before access to synchrotron beamtime, and again there was no discernible oxidation of phase-A during this time. The single-crystal X-ray diffraction study was carried out at the micro-focus macromolecular MX2 beamline of the Australian Synchrotron^13^. Crystal data and details of data collection and refinement are given in Table 1. The crystal was maintained at 100(1) K in an open-flow nitrogen cryostream during measurement. The crystal structure refinement indicates that the phase has the structural formula (U,Pu)(Al_,_Fe)_3_C_3_. The final model converged to *R*1 of 0.0297 for 288 independent reflections (1747 measured reflections), and chemical formula (U_0.59(5)_Pu_0.41(5)_)(Al_0.54(2)_Fe_0.46(2)_)_3_C_3_ (Table 1).

**Table 1:** Crystal structure refinement details for (U,Pu)(Al,Fe)_3_C_3_.

Crystal data	
Formula	(U_0.59(5)_Pu_0.41(5)_)(Al_0.54(2)_Fe_0.46(2)_)_3_C_3_*
Crystal system, space group	Monoclinic, *C*2/*c*
Temperature (K)	100 (1)
*a*, *b, c* (Å)	3.4111 (7), 5.8987 (12), 17.499 (4)
*β* (°)	90.12 (3)
*V* (Å^3^)	352.10 (12)
*Z*	2
Calculated density (g cm^−3^)	7.480
Radiation type and wavelength (Å)	Synchrotron, λ = 0.71075
*μ* (mm^−1^)	40.498
Crystal dimensions (mm)	0.004 $\times$ 0.004 $\times$ 0.004
Reflections for cell refinement	1018, 2.27–20° 2θ

Data collection	
Crystal description	Black triangular wedge
Diffractometer	Dectris EigerX 16 M
Scan type	φ scan with frame widths of 0.1°; counting time per frame of 0.01 s
Beam size (with at half maximum)	~ 20 µm
θ (°) range	2.328–24.692°
Indices range of *h*,* k*,* l*	*h*: ± 4, *k*: ± 6, *l*: ± 20
Absorption correction	Multi-scan SADABS (Bruker, 2001)
*T*_min_, *T*_max_	0.2854, 0.4342
No. of measured, independent and observed [*I* > 2σ(*I*)] reflections	1747, 288, 213
*R*_int_	0.0601
Data completeness to 24.692° θ (%)	100

Refinement	
Number of reflections, parameters, restraints	288, 37, 0
*R*_*1*_[*F*^2^ > 2σ(*F*^2^)], *R*_*1*_(all)	0.0298, 0.0367
*wR*_*2*_[*F*^2^ > 2σ(*F*^2^)], *wR*_*2*_(all)	0.0758, 0.0787
*GoF* (*F*^2^)	0.947
Δ*ρ*_min_, Δ*ρ*_max_ (e Å^−3^)	− 1.34, 1.24

Data processing	
The intensity data sets were processed using XDS^38^, XPREP^39^ and SADABS^40^, finding 1747 reflections with an *R*_int_ of 0.0601. Atomic positions were located using SHELXT-2018/3^41^, and then refined anisotropically using SHELXL-2018/3^42^ using neutral atomic scattering factors	

*U/Pu ratio could not be refined from XRD and was determined from semi-quantitative EDS analysis from numerous points on the phase of interest (see SI dataset 2).

The measured crystal is monoclinic, space group *C*2/*c* with unit-cell parameters: *a* = 3.4111(7) Å, *b* = 5.8987(12) Å, *c* = 17.499(4) Å, β = 90.12(3)°,* V* = 352.10(12) Å^3^, and *Z* = 2. The sharp diffraction spots and low *R*1 indicate a high degree of crystallinity. The mosaicity (full-width-half-maximum of a diffraction peak) was explored to further characterise crystallinity. The average mosaicity is 0.31°, with a standard deviation of 1.03° influenced by a minority of high mosaicity reflections. Out of 1747 measured reflections, 109 have mosaicity > 1°, of which 31 have mosaicity values between 2 and 10°, and 7 extreme outliers have mosaicity > 19°. This pattern may arise from highly anisotropic radiation damage, that leaves the overall crystal highly ordered.

**Figure 2:**
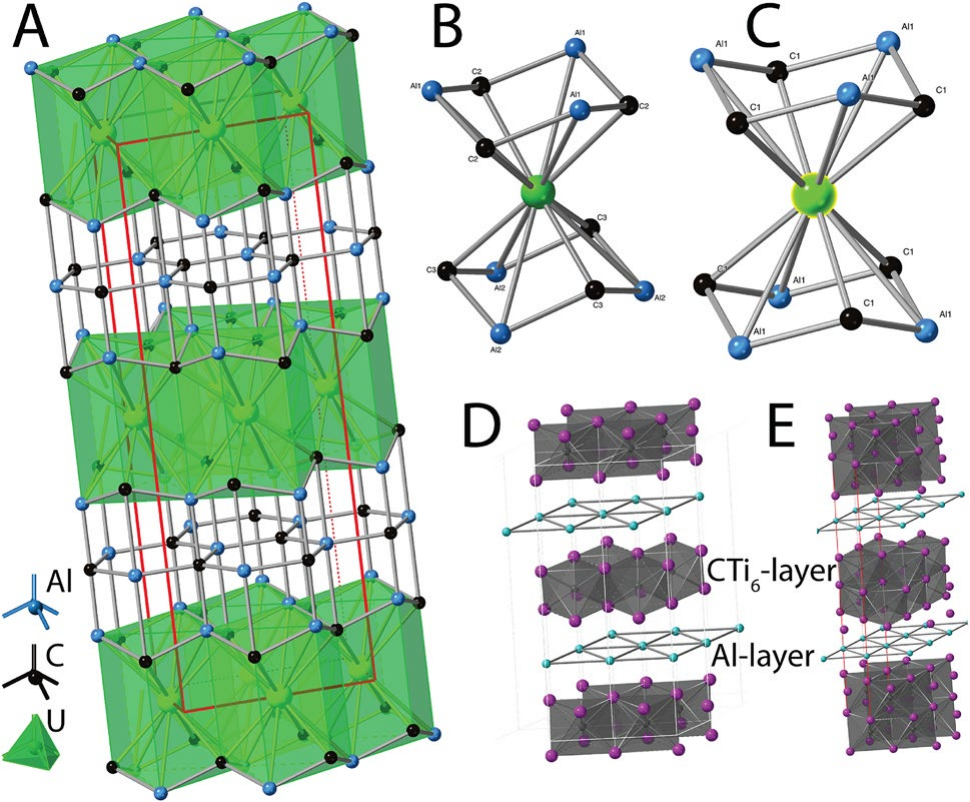
Crystal structure of (U,Pu)(Al,Fe)_3_C_3_ discovered in a hot particle from Maralinga, and relations to MAX phases. (**A**) Overview of the layered structure of (U,Pu)(Al,Fe)_3_C_3_. (**B**, **C**) Coordination of the actinide sites in (U,Pu)(Al,Fe)_3_C_3_. (**B**) and in the topologically similar UAl_3_C_3_ (**C**). The derivative MAX structure of the new phase (**A**) is compared to two MAX phase sensu stricto structures (Al(Ti)_n+1_Ci_n_): (**D**) Ti_2_AlC (n = 1), Cambridge Crystallographic Data Centre deposition number 1681503); (**E**) Ti_3_AlC_2_ (n = 2), International Crystal Structure Database #93503).

The crystal structure consists of alternating layers of face-sharing, [6 + 6]-coordinated [(U,Pu)C_4_(Al,Fe)_4_] polyhedra, and sheets of ‘graphite-like’ hexagonal C_3_(Al,Fe)_3_ rings (Fig. 2). This topology is similar to UAl_3_C_3_^14^. Both compounds have one metal site; however, the crystal structure of the Pu-bearing phase is monoclinic (*C*2/*c*) with two C and two Al sites, whereas UAl_3_C_3_ is hexagonal (*P*6_3_*mc*) with three C and three Al sites.

UAl_3_C_3_ and (U,Pu)(Al_,_Fe)_3_C_3_ are related to a family of ternary carbides and nitrides called “*MAX phases*”, named from their general formula of M_n+1_AX_n_ (A is an A group (generally IIIA and IVA) element; M is a metal, typically an early transition metal or a REE; X is C or N)^15–17^. MAX structures are usually hexagonal (*P*6_3_/*mmc*), and comprise alternating M_6_X octahedral- and Al-layers (Fig. 2D,E); the octahedral layers can have thicknesses of n = 1 (Fig. 2D), n = 2 (Fig. 2E), up to n = 6 octahedra.

(U,Pu)(Al,Fe)_3_C_3_ and UAl_3_C_3_ are “*derivative-MAX phases*”, with the general formula (MC)_n_(Al_3_C_2_)_m_ and n = m = 1. Derivative-MAX phases contain metal ions (M) in [6 + 6]-fold coordination (e.g., MC_6_Al_6_), compared to sixfold coordination in MAX phases. As a result, derivative-MAX phases frequently contain high-Z metals on the M-site, including Y, Gd-Tm, Yb, Lu and U^14,18,19^. The Al layers in MAX phases can be described as centred hexagonal rings, but the Al_3_C_3_ layers in derivative MAX phases lack centring.

In MAX phases, reduction in symmetry from hexagonal to monoclinic occurs either via ordering on the M-site, resulting in a super-cell, e.g., (V_0.67_Zr_0.33_)_2_AlC is monoclinic with a 3*a* supercell^20^; or via lattice distortion caused by mixing another ion on the A-site, e.g. Cu in Ti_3_(Al_1−δ_Cu_δ_)C_2_^21^. In (U,Pu)(Al,Fe)_3_C_3_, the reduction in symmetry from hexagonal to monoclinic arises from the incorporation of Fe on the Al-dominant A-site, and is reflected by changes in the coordination around the M-site actinide atoms (Fig. 2B,C; Table 2). Overall, the bond lengths are slightly longer around the (U,Pu) site in (U,Pu)(Al,Fe)_3_C_3_; in particular, the shortest (U,Pu)-C distance is 2.603(15) Å in (U,Pu)(Al,Fe)_3_C_3_, but only 2.491 Å in UAl_3_C_3_. The actinide coordination is also more anisotropic in the new compound, with 6 versus 3 distinct distances (Table 2).

**Table 2:** Coordination around the (U,Pu) and U sites in (U,Pu)(Al,Fe)_3_C_3_ and UAl_3_C_3_.

	(U,Pu)(Al,Fe)_3_C_3_		UAl_3_C_3_	
1	Pu–C1	2.603 (15)	U–C3	2.49067
2	Pu–C1	2.603 (15)	U–C3	2.49067
3	Pu–C1	2.616 (16)	U–C3	2.49067
4	Pu–C1	2.616 (16)	U–C2	2.60074
5	Pu–C1	2.627 (15)	U–C2	2.60074
6	Pu–C1	2.627 (15)	U–C2	2.60074
		**2.615 (15)**		**2.55 (6)**
7	Pu–Al1	3.055 (4)	U–Al2	3.03388
8	Pu–Al1	3.055 (4)	U–Al2	3.03388
9	Pu–Al1	3.058 (4)	U–Al2	3.03388
10	Pu–Al1	3.058 (4)	U–Al1	3.07126
11	Pu–Al1	3.067 (4)	U–Al1	3.07126
12	Pu–Al1	3.067 (4)	U–Al1	3.07126
		**3.060 (4)**		**3.05 (2)**
13	Pu–Pu	3.4070 (5)	U–U	3.389
14	Pu–Pu	3.4070 (5)	U–U	3.389
15	Pu–Pu	3.4070 (5)	U–U	3.389
16	Pu–Pu	3.4070 (5)	U–U	3.389
17	Pu–Pu	3.4111 (7)	U–U	3.389
18	Pu–Pu	3.4111 (7)	U–U	3.389
		**3.4083 (6)**		**3.389**

Average values are in bold.

## Origin of derivative-MAX phase at Maralinga

This is the first reported occurrence of a (U,Pu) carbide associated with nuclear testing or nuclear incident. Carbon-rich; Fe, Al, Cs-bearing; but U/Pu-free particles were generated during the 2011 Fukushima nuclear accident; these particles, however, are amorphous^22^. To date, the only reported occurrence of a crystalline U-carbide is in the form of minor UC within particles resulting from the impact of depleted U ammunition on armoured vehicles during the 1999 Balkan conflict (Kosovo) and the 1991 Gulf war (Kuwait)^23^. The contrast in crystal structure^24^ and composition and antithetical conditions for their formation indicate that these occurrences are largely irrelevant to the Maralinga problem.

The finding of these derivative-MAX phases poses two questions: how did they form; and why are they still present after almost 60 years of exposure to the environment? The starting point for their origin is their composition and the sourcing of Al, Fe, and C within the steel-structures (known as featherbeds) used to support the Vixen-B tests (Fig. 3.7 in MARTAC report^25^). Although large quantities of the products of the explosion would exclude air, the many studies of other particles from sub-critical nuclear incidents (e.g.^2,26^) have not identified Pu or U carbides, suggesting that the various explosives will not supply enough carbon to form such a phase. The UC phase^24^ identified by Lind et al.^23^ has been shown to form during impact of depleted uranium ammunition; these authors do not speculate on the origin of carbon, but the composite armour is the most likely source, since it can include plastics or SiC. In the case of Bruce, the carbon source is most likely from the paraffin wax used in some of the featherbeds for the Vixen-B tests (Figs. 3.7 & 3.8 in MARTAC report^25^). This paraffin would facilitate the formation of a C-rich, reducing environment conducive to the formation of C-saturated materials.

Although the bulk Bruce composition provides the elements of the derivative-MAX phase, the problem of their combining to form crystalline particles remains. Since other elements must be prevented from contaminating the fissile material, it is unlikely that they would be in sufficient proximity to the Pu “pit” (hollow sphere) to combine with it during the implosion phase that would have been the objective of these subcritical tests. Burns et al.^7^ noted that during the Vixen B trials, “the plutonium melted and burned as it was ejected vertically to heights of up to 800 m and dispersed by the prevailing winds”. Particles experienced temperatures well in excess of 2000˚C, as demonstrated for example by evidence of (Pu,U)O_2_ vapor coexisting with (Pu,U)O_2_ solid in other particles^6^. These temperatures are consistent with the high-T synthesis of MAX and derivative-MAX-type phases^15^, which are typically produced at temperatures between ~ 1500 and 1600 °C under a reducing atmosphere. Gesing et al.^18^ prepared the closely related UAl_3_C_3_ by arc melting (~ 3500 °C) pellets of the constituent elements in the correct proportions in an argon atmosphere. They produced 25 × 100 × 200 µm^3^ crystals, but these samples reacted with the humidity in the air and decomposed within a few hours. Bai et al.^27^ prepared a mixture of UAl_3_C_3_ and U_2_Al_3_C_4_ by sintering UC, Al and C at 1200–1400 °C for 2 h under an Ar atmosphere. They did not comment on the stability of their product. However, insofar as Pu metal is highly pyrophoric, with particles even in the tens of microns size spontaneously igniting in air, it is highly unlikely that the derivative-MAX phase containing Pu would form from the particles in the plume.

An alternative is its formation during the compression phase, in which case the high pressure might facilitate the reactions and have a role in stabilising the (U,Pu)(Al_,_Fe)_3_C_3_ phase. Some materials, notably nanoscale diamond powder, can be prepared via explosive synthesis^28^. However, in this case, high pressure does not appear to be a requisite component: closely related derivative-MAX phases were prepared in the laboratory at ambient pressure^15,27^, and the fact that the (U,Pu)(Al_,_Fe)_3_C_3_ phase formed via eutectic precipitation from Melt2 (Fig. 1) suggests that temperature is the dominant variable. Where pressure could be a factor in the outcome, however, is in the fissile material after its maximum compression. In the absence of nuclear reactions even a miniscule instability would eject droplets, even from a jet of liquid metal at very high velocity. These micro-droplets would pierce the featherbed, accumulating, melting, mixing, and dissolving its elements, emulating arc melting instead of a solid phase reaction. The highly ordered microcrystals of (U,Pu)(Al_,_Fe)_3_C_3_ then form via eutectic crystallisation from the resulting complex polymetallic melt. The brief time before it solidifies accounts for the small crystal size, and reaction with air is prevented by the outer layer of metal on the particles. The high temperature stability of the derivative-MAX phase is responsible for early crystallisation, which enables it to scavenge the majority of Pu present in the melt, and it inhibits its decomposition from the Pu + O_2_ reactions in the plume during the brief interval before they cool down.

## Environmental stability and contribution to slow radionuclide release at Maralinga

The (U,Pu)(Al,Fe)_3_C_3_ phase in this study has been remarkably stable under ambient conditions for more than 60 years, despite the fact that U and Pu are in low valence state as carbides, as confirmed by the XANES data collected on the Bruce particle^6^. The metallic Al–Fe matrix surrounding this phase may have assisted in maintaining reducing conditions, but the crystal structure of (U,Pu)(Al,Fe)_3_C_3_ also appears to be able to retain actinides effectively. The phase and its uniquely low valence Pu and U were stable in contact with atmosphere over the 3 months of direct exposure, even in micro-crystalline state—e.g., the FIB cut extracted in June 2022 was stable for 3 months before it was measured at the synchrotron in Oct 2022.

The diffraction pattern showed high degree of crystallinity indicating that the structure remained intact, despite evidence of radiation damage in the form of physical cracks in the phase-A regions (Fig. 1C). According to the empirical formula (U_0.59(5)_Pu_0.41(5)_)(Al_0.54(2)_Fe_0.46(2)_)_3_C_3_, the crystal would have experienced ~ 9.2 × 10^24^ alpha decay events/m^3^ over 60 years, based on a density of 7.48 g/cm^3^, with an approximate isotopic composition (see SI 1) of ~ 95% ^239^Pu +  ~ 5% ^240^Pu and ~ 93% ^235^U +  ~ 6% ^238^U +  ~ 1% ^234^U and specific activities of ^239^Pu = 2.3 × 10^12^ Bq/kg, ^240^Pu = 8.4 × 10^12^ Bq/kg, ^235^U = 8.00 × 10^7^ Bq/kg, ^238^U = 1.24 × 10^7^ Bq/kg and ^234^U = 2.31 × 10^11^ Bq/kg. This is comparable to the dose of 2.1 × 10^25^ α-decay events/m^3^, which caused a monoclinic zirconolite doped with 4 mol% ^238^PuO_2_^29^ to become amorphous, and reflects the high radiation resistance of this material.

Knowledge of the actinide valence state and crystal chemistry underpins predictions of the behaviour of the material as it is subjected to irradiation and environmental agents^30^. In general, MAX phases are noted for their extraordinary mechanical, physical and chemical properties, including outstanding resistance to oxidation and corrosion and higher radiation damage tolerance compared to refractory binary transition metal carbides^16,17,31–33^. Titanium–Al–C and Ti–Si–C compounds in particular maintain crystallinity even after irradiation at temperatures up to ~ 700 °C, though there was evidence of radiation damage, including anisotropic swelling. At higher temperatures there was evidence of dynamic recovery. As a result, MAX-phase have found uses in many high temperature applications, and are being considered for use as cladding material in nuclear reactors^15,17,27,34^.

At Maralinga, the activities of ^239^Pu found in animals (e.g., 4.1 × 10^–3^ to 2.1 Bq/kg in mammals) have been approximately constant for nearly 30 years^35^. This steady-state uptake appears surprising at first glance since semi-arid environments are marked by infrequent but high intensity rain events that have the potential for rapid release of soluble or colloidal radionuclides. However, this may be explained by the presence of refractory Pu-hosting phases in the hot particles from Maralinga. As a result of their complex makeup and relative chemical stability, these particles are subjected to slow mechanical and chemical weathering, contributing to the on-going but slow release of colloidal plutonium into the environment. An important corollary observation relevant to actinide environmental chemistry in general is that unusual, high energy conditions can produce compounds and chemical species that fall totally out of the bounds of the equilibrium oxyhydroxide species that should form quite quickly. However, their stability in contact with air and moisture actually renders them less hazardous over decades to centuries than more typical materials.

## Implications for nuclear waste disposal

The discovery of REE-bearing MAX phases led Wang et al.^17^ to question whether it was possible to incorporate U and/or Pu and use these MAX phases for nuclear waste disposal. The observed environmental stability of a Pu-rich derivative-MAX phase at Maralinga provides direct evidence that these structures, in conjunction with a metallic matrix, could be a potential phase for actinide immobilization. This is even though the (U,Pu)(Al,Fe)_3_C_3_ phase contains low valence Pu/U, that is usually highly reactive in oxidizing or hydrous environments, as demonstrated for example by the fast oxidation of metallic U that remains in particles resulting from use of depleted uranium ammunition^36^. Requirements for actinide immobilization include high chemical durability, low solubility, a structure resistant to radiation damage, and avoid criticality^3,17,29,37^. The Maralinga particles show that polymetallic melts may cool down into materials with phase compositions and microtextures that fulfil most of these requirements.

Hence, our findings suggest a new possible pathway for geological disposal of high-grade Pu waste, via direct conversion of large pieces of Pu metal, such as retired Pu-pits, to a suitable waste form. The current process is oxidation to PuO_2_^41^, typically in solution. While the solid-state reaction is difficult to take to completion, the oxidative dissolution is highly exothermic, releases large quantities of gas, and runs the risk of criticality events. If these inert derivative-MAX phases do form spontaneously from a mixture of Pu, Fe, Al, and C initiated by melting, a durable waste form would be produced in a single melting step followed by rapid quenching; such a process may involve a greatly reduced degree of hazard, and requires addition of only cheap and benign materials (Fe, Al, C), compared for example to Synroc^41^.

### Supplementary Information

Supplementary Information 1.

Supplementary Information 2.

Supplementary Information 3.

## Data Availability

The cif data have been deposited with the Cambridge Crystallographic Data Centre (CCDC deposition number 2332998), a repository for electronic crystallographic data (https://www.ccdc.cam.ac.uk); and are also available in the supplementary information.
