# Supplementary material for: Environmental stability of a uranium-plutonium-carbide phase
Source: Sci Rep. 2024 Mar 17;14:6413. doi: 10.1038/s41598-024-56885-7 (PMC10944826; doi:10.1038/s41598-024-56885-7)
Supplement: Supplementary file 1 — Supplementary Information 1. [file 41598_2024_56885_MOESM1_ESM.pdf]

FROM : PHONE NO. : P01

Dr G. Williams ARL
As promised
Nick Chaplette(?)
6/6/95

D/Sc(Nuc)2/5/6/10

28 March 1985

available in DRE if required.

MINOR TRIALS SCHEDULE

The following unclassified tabulation is extracted from classified material which has been provided for the use of Australian officials who may be granted access to UK information at the classification level which it carries. The three classified documents are referenced MTS(60)VB, MTS(61)VB and MTS(63)VB all dated 15 March 1985 and originate from Science(Nuclear)2.

Extract from MTS(60)VB

Date	Round No	Site	Quantity	Met Info	Composition
		Taranaki			
29 Aug	Calibration	Pad B	Pu, U^{235}, U^{238} and Beryllium See Below	See AWRE Report No T4/61	See Below
8 Sep	VB AH	Pad D			
29 Sep	VB DH	Pad C			
3 Oct	VB EH	Pad E			

Extract from MTS(61)VB

Date	Round No	Site	Quantity	Met Info	Composition
		Taranaki			
-	Calibration	Pad L	Pu, U^{235}, U^{238} and Beryllium See Below		See Below
13 Apr	C	Pad F		15kn 170°	
23 Apr	D	Pad J		8kn 165°	
8 May	B	Pad G		9kn 190°	
18 May	E	Pad H		10kn 190°	
25 May	G	Pad A			

Not as per [illegible] TA[illegible] 1987

Extract from MTS(63)VB

Date	Round No	Site	Quantity	Met Info	Composition
		Taranaki			
19 Mar	5301/1	Pad K	Pu, U^{235}, U^{238} and Beryllium See Below		See Below
26 Mar	5301/2	Pad PD		15kn 170°	
2 Apr	5301/3	Pad PE		10kn 190°	
9 Apr	5301/4	Pad PC		8kn 165°	
14 Apr	5301/5	Pad M		10kn 190°*	

*Records conflict 190° is best estimate

Quantity of Material

Total Plutonium (including 1% Gallium alloying agent)	22.2 kg
Total U^{235} (all of composition close to data below)	22.4 kg
Total U^{238} (assume natural uranium)	24.9 kg approx
Total Beryllium	17.6 kg approx

Composition of Materials

Plutonium - Bulk* of material	Pu^{239}	~95%)		
	Pu^{240}	~5%)	of Pu	
	Pu^{241}	~0.5%)		

*The Pu^{240} content varied from 1 to 5%

	Pu	~99%)	
	Ga	1%)	of alloy
	Am	0.1%)	

U^{235} - Typical figures	U^{235}	~93%)	
	U^{234}	1%)	
	U^{236}	0.3%)	of Uranium
	U^{238}	~6%)	

Science(Nuclear)2
Ministry of Defence
Main Building
Whitehall
London SW1A 2HB
